# PHYSICAL AND MENTAL HEALTH OUTCOMES AMONG OLDER MILITARY VETERANS

**DOI:** 10.1093/geroni/igac059.596

**Published:** 2022-12-20

**Authors:** Scott Landes, Janet Wilmoth

## Abstract

Older veterans are a unique health population, with physical and mental health outcomes impacted by the positive health aspects of military social capital as well as the negative aspects of military-related hazards. This symposium focuses on physical and mental health outcomes among older military veterans both before COVID-19, and during the COVID-19 pandemic. Three studies address veteran health outcomes pre-pandemic. Two of the pre-pandemic studies focus on veteran-only samples in order to determine whether aspects of marital quality predicted levels of loneliness, and risk factors for trauma re-engagement among those with medical illness. The third pre-pandemic study examines whether the increased mortality risk observed among older veterans compared to nonveterans varies by combat status. Two studies address veteran health outcomes during the COVID-19 pandemic. The first uses a sample of older veterans with PTSD who were surveyed pre-pandemic and during the pandemic in order to ascertain the mental and physical health impact of the pandemic. The second COVID-19 study uses data from a study of Veterans Affairs’ Home Based Primary Care (HBPC) providers to learn about the best practices discovered to ensure COVID-19 vaccination uptake by some of the most vulnerable older veterans. Results from each of these studies will shed light on policies and practices needed to ensure the best physical and mental health outcomes for older military veterans.

